# Supplementary material for: Synthesis, Photoswitching Behavior and Nonlinear Optical Properties of Substituted Tribenzo[a,d,g]coronene
Source: Molecules. 2023 Feb 2;28(3):1419. doi: 10.3390/molecules28031419 (PMC9919552; doi:10.3390/molecules28031419)
Supplement: Supplementary file 1 [file molecules-28-01419-s001.zip › molecules-2146297-supplementary.pdf]

Synthesis, Photoswitching Behavior and Nonlinear Optical Properties of Substituted Tribenzo[a,d,g]coronene

Xueqing Li[1,†], Jie Zhao[2,†], Wei Wang[1], Yiming Li[1], Yunfei Li[2,*], Shuyun Zhou[2] and Jinchong Xiao[1,*]

[1] Key Laboratory of Chemical Biology of Hebei Province, College of Chemistry and Environmental Science, Hebei University, Baoding 071002, P. R. China. E-mail: jcxiaoicas@163.com

[2] Key Laboratory of Photochemical Conversion and Optoelectronic Materials, Technical Institute of Physics and Chemistry, Chinese Academy of Sciences, Beijing 100190, P. R. China. E-mail: liyunfei@mail.ipc.ac.cn

Contents

Figure S1-S3 ········ S2

Figure S4-S6 ········ S3

Figure S7-S9 ········ S4

Figure S10-S12 ········ S5

Figure S13-S15 ········ S6

Figure S16-S18 ········ S7

Table S1 ········ S8

Figure S19 and Table S2 ········ S9

Table S3 ········ S18

Figure S20 and S21 ········ S19

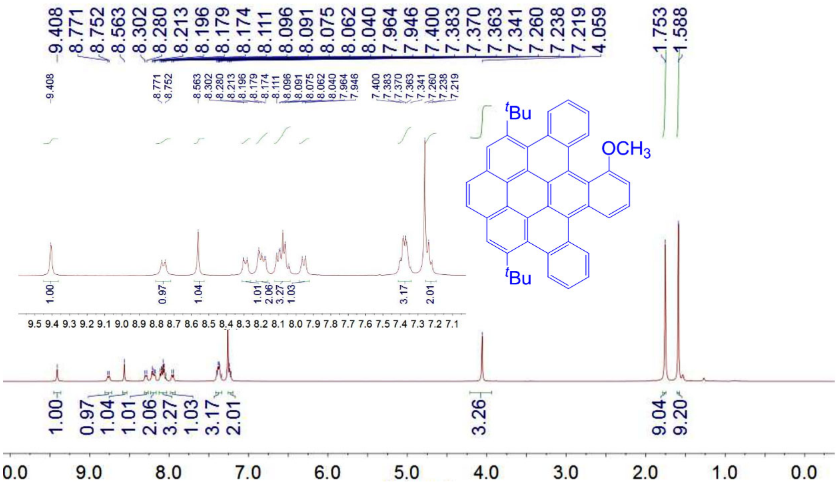

Figure S1 ^{1}H NMR spectrum of **3a**.

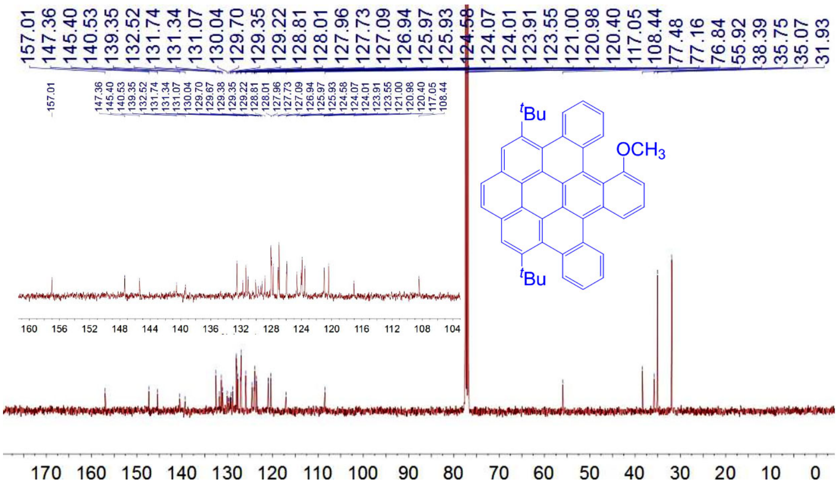

Figure S2 ^{13}C NMR spectrum of **3a**.

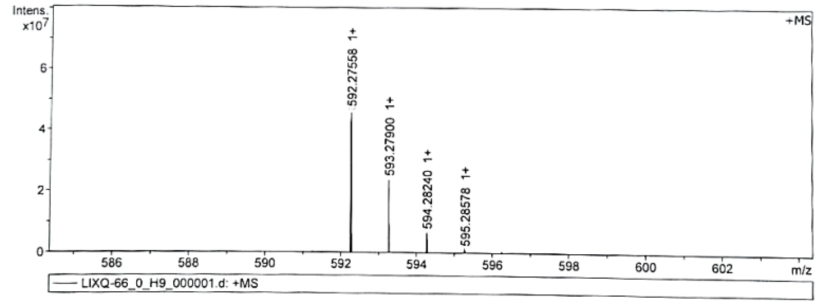

Meas. m/z	#	Ion Formula	Score	m/z	err [ppm]	Mean err [ppm]	mSigma	rdb	e⁻ Conf	N-Rule
592.275577	1	C45H36O	100.00	592.276067	0.8	0.8	14.1	28.0	odd	ok

Figure S3 HR-MS spectrum of **3a**.

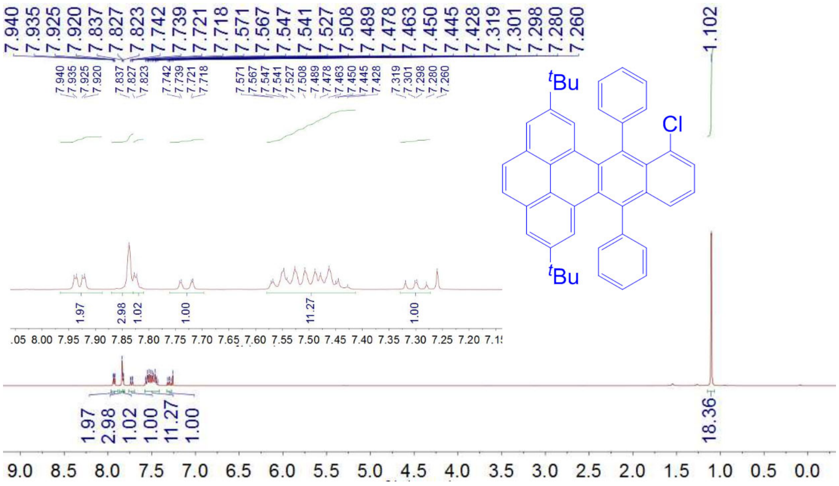

Figure S4 ^{1}H NMR spectrum of **2b**.

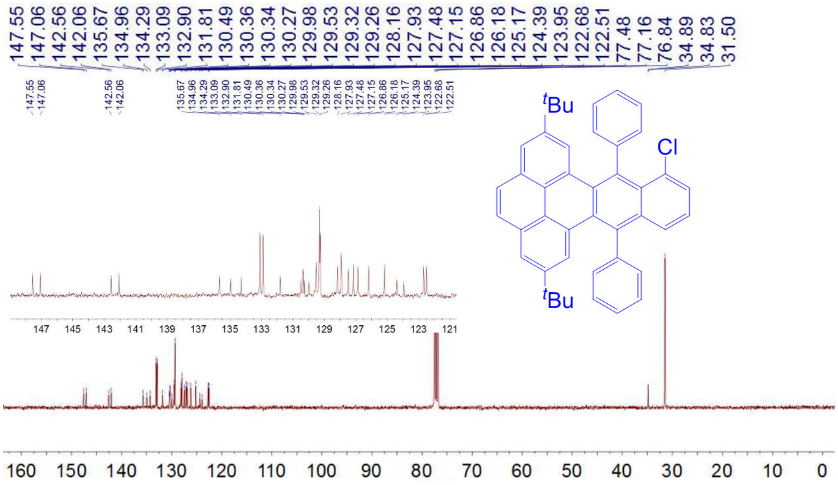

Figure S5 ^{13}C NMR spectrum of **2b**.

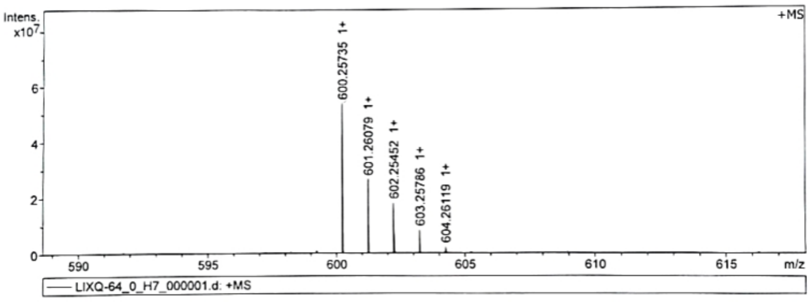

Meas. m/z	#	Ion Formula	Score	m/z	err [ppm]	Mean err [ppm]	mSigma	rdb	e⁻ Conf	N-Rule
600.257352	1	C44H37Cl	100.00	600.257830	-0.8	0.9	167.0	26.0	odd	ok

Figure S6 HR-MS spectrum of **2b**.

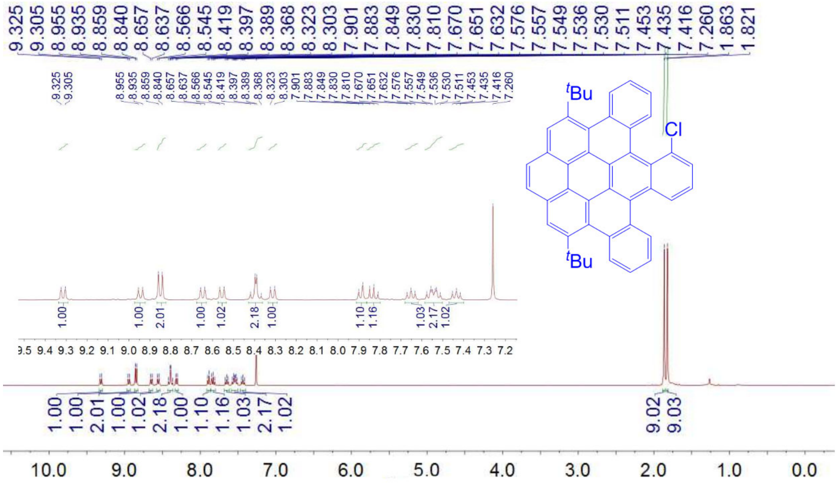

Figure S7 ^{1}H NMR spectrum of **3b**.

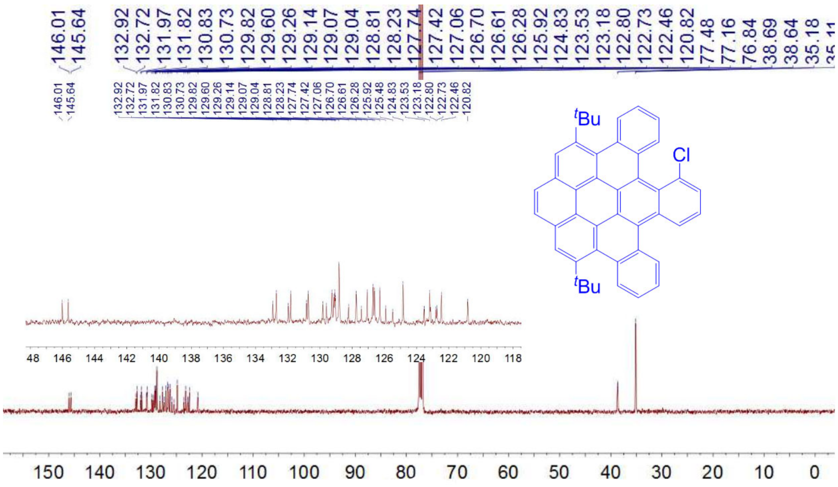

Figure S8 ^{13}C NMR spectrum of **3b**.

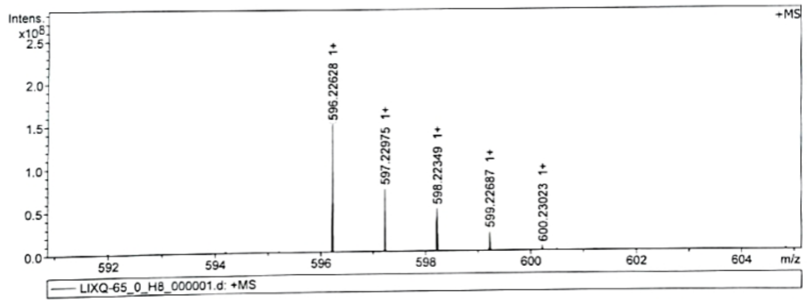

Meas. m/z	#	Ion Formula	Score	m/z	err [ppm]	Mean err [ppm]	mSigma	rdb	e⁻ Conf	N-Rule
596.226278	1	C44H33Cl	100.00	596.226530	-0.4	0.5	166.6	28.0	odd	ok

Figure S9 HR-MS spectrum of **3b**.

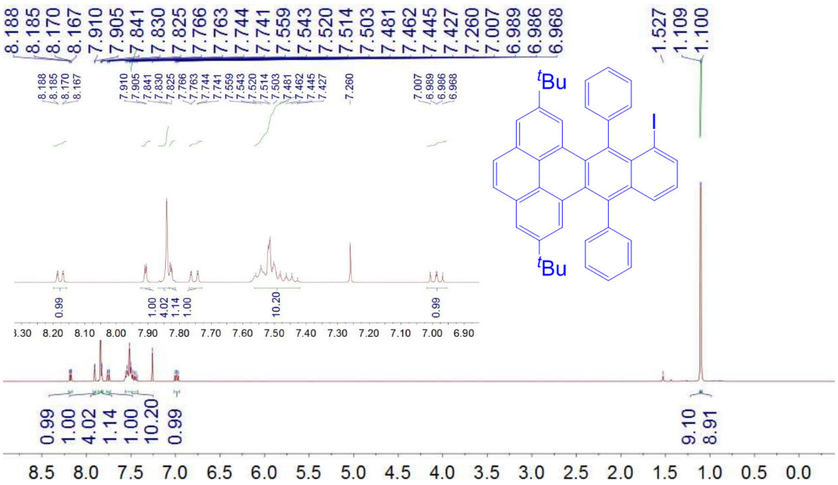

Figure S10 ^{1}H NMR spectrum of **2d**.

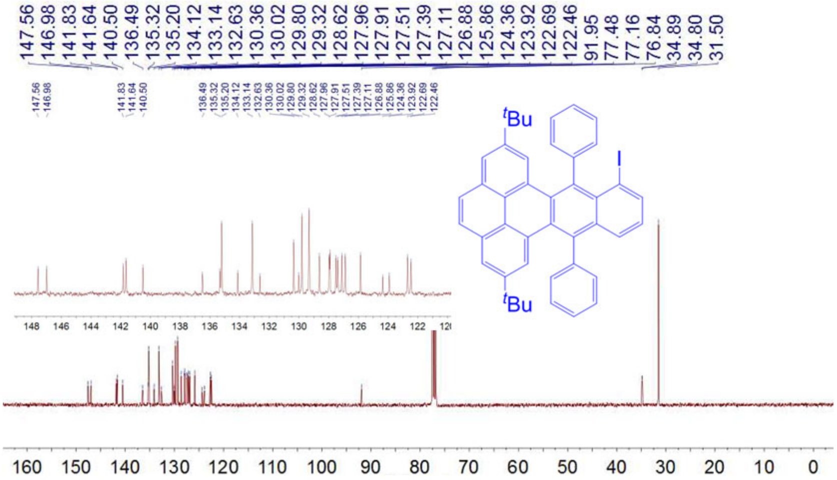

Figure S11 ^{13}C NMR spectrum of **2d**.

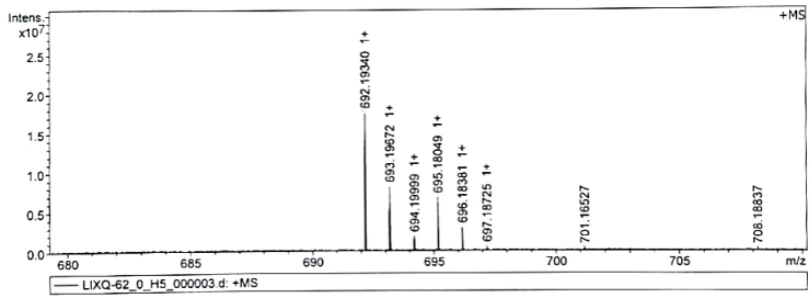

Meas. m/z	#	Ion Formula	Score	m/z	err [ppm]	Mean err [ppm]	mSigma	rdb	e⁻ Conf	N-Rule
692.193396	1	C44H37I	100.00	692.193451	-0.1	0.1	7.7	26.0	odd	ok

Figure S12 HR-MS spectrum of **2d**.

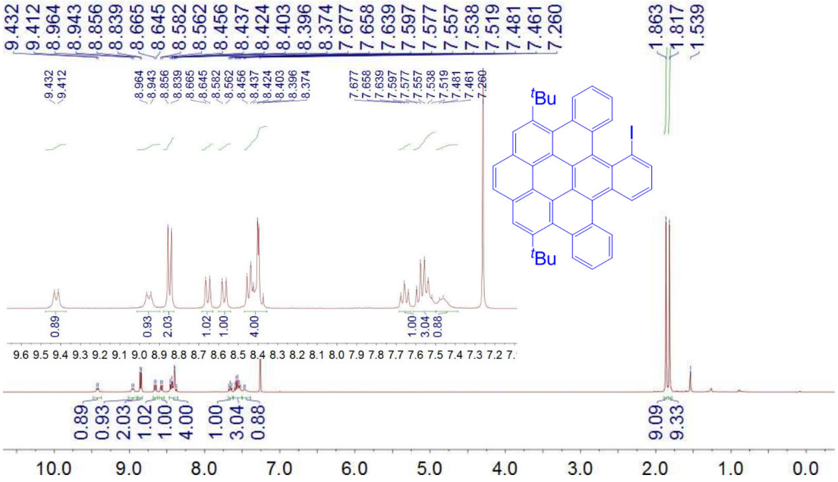

Figure S13 ^{1}H NMR spectrum of **3d**.

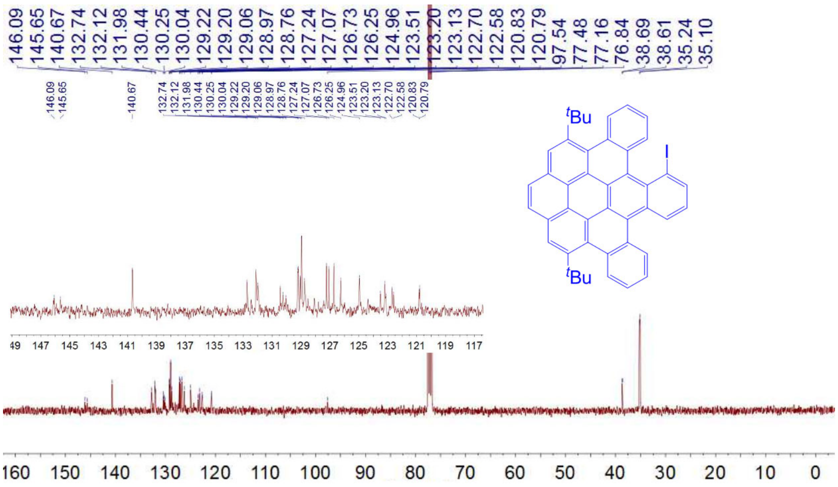

Figure S14 ^{13}C NMR spectrum of **3d**.

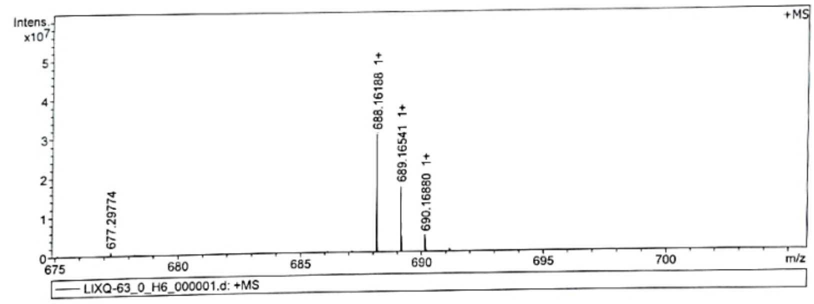

Meas. m/z	#	Ion Formula	Score	m/z	err [ppm]	Mean err [ppm]	mSigma	rdb	e⁻ Conf	N-Rule
688.161882	1	C44H33I	100.00	688.162150	0.4	0.3	40.3	28.0	odd	ok

Figure S15 HR-MS spectrum of **3d**.

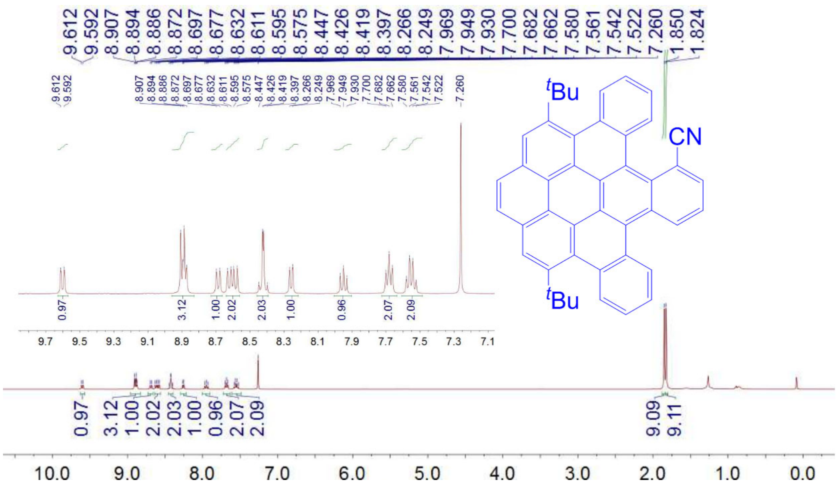

Figure S16 ^{1}H NMR spectrum of **3e**.

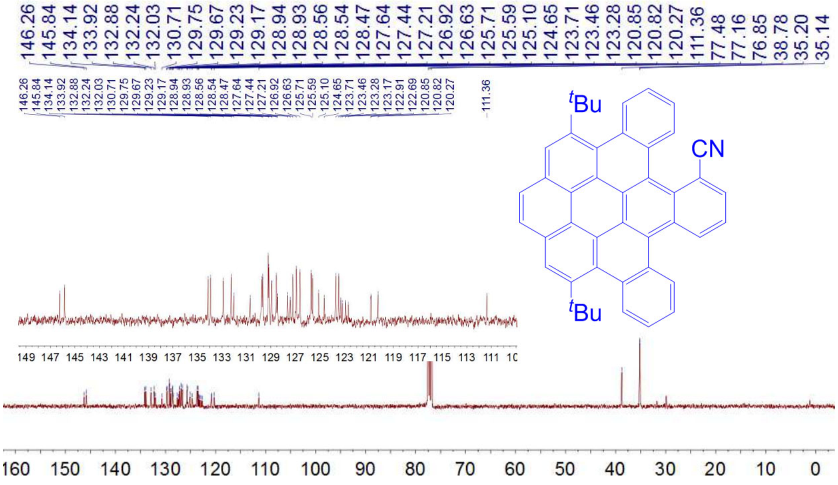

Figure S17 ^{13}C NMR spectrum of **3e**.

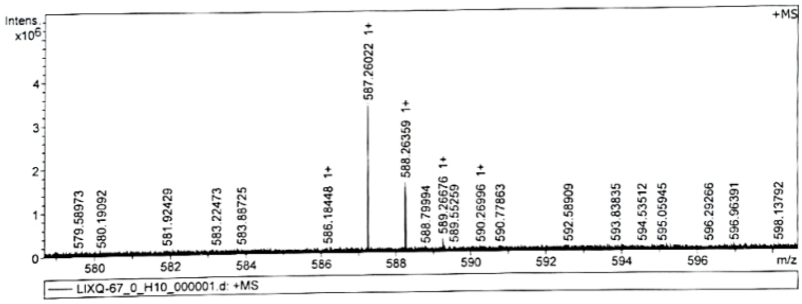

Meas. m/z	#	Ion Formula	Score	m/z	err [ppm]	Mean err [ppm]	mSigma	rdb	e⁻ Conf	N-Rule
587.260217	1	C45H33N	100.00	587.260751	0.9	0.9	18.8	30.0	odd	ok

Figure S18 HR-MS spectrum of **3e**.

Table S1 Crystal data and structure refinement for **3b**.

	**3b**
Empirical formula	$C_{44}H_{33}Cl$
Formula weight	596.23
Temperature/K	298(2)
Crystal system	Monoclinic
Space group	C2/c
Unit cell dimensions	a = 26.684(3)Å<br>b = 13.5714(13Å<br>c = 19.003(2)Å<br>α = 90°<br>β = 100.020(4)°<br>γ = 90°
Volume/$Å^3$	6776.8(12)
Z	8
Density/(g/cm^3)	1.171
Absorption Coefficient/mm^{-1}	0.142
F(000)	2512
Crystal size/mm^3	0.28×0.17×0.06
Radiation type	Mo K/a<br>λ = 0.71073
CCDC number	2232268

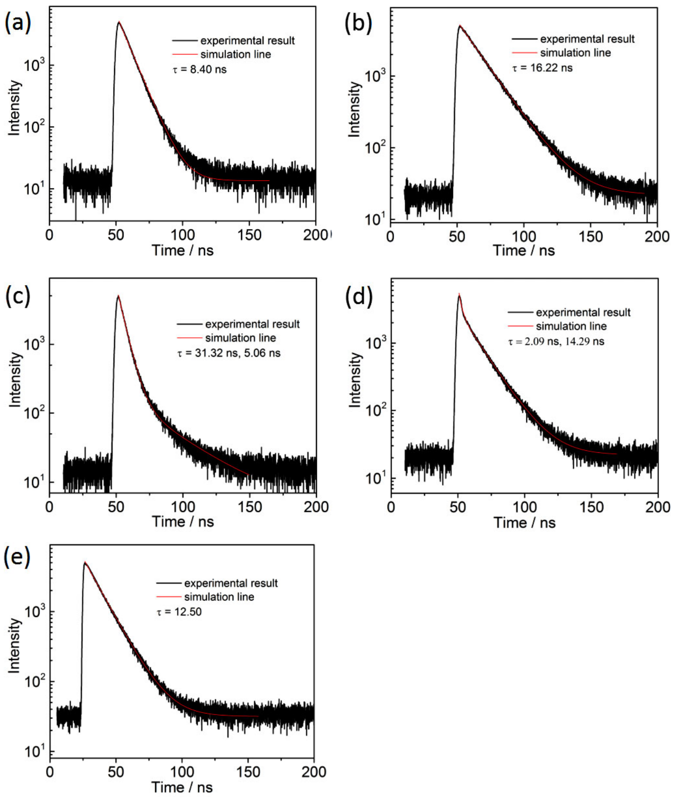

Figure S19 Fluorescence decay of (a) **3a**, (b) **3b**, (c) **3c**, (d) **3d**, (e) **3e** in degassed dichloromethane. The excitation wavelength is 455 nm.

Table S2 Cartesian coordinates of optimized **3a-3e**

3a

C	2.14051480	-3.62098387	-0.52025083
C	0.75291323	-3.47749718	-0.28408566
C	0.23880125	-2.16546463	-0.12580457
C	1.13810793	-1.07246096	0.00622341
C	2.54140733	-1.30482188	0.03253380
C	3.04550925	-2.56793129	-0.45980910
C	-0.15434734	-4.58481356	-0.27891350
C	-1.50038032	-4.39140946	-0.10722000
C	-2.04370718	-3.07276644	0.01779468
C	-1.17036317	-1.95798215	-0.04801790
C	-3.40713488	-2.82805865	0.30601745
C	-3.96062177	-1.55711296	0.39707589
C	-3.12304640	-0.45335208	-0.02404892
C	-1.71388119	-0.64344258	-0.06399647
C	-0.81189939	0.49067158	-0.08697745
C	0.60487544	0.27219918	0.08452724
C	-1.32361591	1.79935586	-0.18450090
C	-0.43778418	2.92395915	0.02845763

C	0.97726046	2.70236887	0.07719103
C	1.47960304	1.36237032	0.26533487
C	-0.91917772	4.25260538	0.22243871
C	-0.05489402	5.32496783	0.25209384
C	1.32114268	5.13883682	0.00535346
C	1.82817805	3.85208477	-0.11158391
C	2.78320705	1.04588825	0.84838197
C	-2.69937622	1.93302273	-0.66493937
C	3.44337412	1.95244713	1.71701838
C	4.58717436	1.60140882	2.41232112
C	5.10885765	0.30227798	2.28576454
C	4.46729179	-0.61241526	1.46897073
C	3.31214242	-0.27351500	0.72136008
C	-3.58887365	0.81539313	-0.57516008
C	-4.84080816	0.89487262	-1.23251525
C	-5.25031735	2.02992388	-1.91093577
C	-4.38490132	3.13205903	-1.99178861
C	-3.13524202	3.06951912	-1.39753319
C	-5.35155243	-1.45229179	1.10842658
C	-5.28949207	-2.32630735	2.39416969
C	-6.49457449	-2.00392391	0.22589314
C	-5.70283849	-0.03455810	1.61194844
O	3.11196463	3.59261718	-0.45718929
C	4.02412456	4.65267603	-0.62371115
C	4.45625720	-2.81889454	-1.08851715
C	4.24461292	-3.52748068	-2.45676930
C	5.32107944	-3.75046998	-0.20883090
C	5.23737178	-1.52906677	-1.42370031
H	2.48878175	-4.61378989	-0.81137234
H	0.24844209	-5.59421337	-0.39973227
H	-2.18152882	-5.24589351	-0.06763047
H	-4.02528135	-3.69992320	0.52880735
H	-1.98299660	4.41706172	0.38012873
H	-0.43843710	6.33349585	0.42536012
H	1.97229241	6.00590740	-0.10002773
H	3.00168498	2.93140988	1.89637800
H	5.05223415	2.31858611	3.09361719
H	5.98479773	-0.00177925	2.86439678
H	4.81835866	-1.64178931	1.44963734
H	-5.47255082	0.01023253	-1.25623955
H	-6.21787998	2.04459014	-2.41882372
H	-4.66889379	4.01787213	-2.56551435
H	-2.44528151	3.89422268	-1.56001961
H	-4.46462711	-2.00719640	3.04999359

H	-6.23114582	-2.22170422	2.95620835
H	-5.15565111	-3.39571768	2.18119532
H	-6.64960141	-1.41016939	-0.68698383
H	-7.44418560	-2.00100855	0.78601650
H	-6.29362811	-3.04073028	-0.08630756
H	-4.87040784	0.40154546	2.18581011
H	-5.96256847	0.66950407	0.81512893
H	-6.57245859	-0.10055732	2.28483423
H	4.98839362	4.19445042	-0.88041137
H	3.71801196	5.33106184	-1.44027733
H	4.14299092	5.24377317	0.30248190
H	3.59790203	-2.92845191	-3.11654731
H	5.21607506	-3.65842461	-2.95951388
H	3.79425248	-4.52483761	-2.35918862
H	5.56905841	-3.30242266	0.76466201
H	6.27199233	-3.98066650	-0.71718769
H	4.80793396	-4.70462519	-0.01158441
H	4.61222217	-0.81949331	-1.98765360
H	5.62456137	-1.00478562	-0.54425118
H	6.10155841	-1.78779538	-2.05599389
**3b**			
C	2.26411095	-3.52750841	-0.52276142
C	0.87032527	-3.42675268	-0.30015441
C	0.31583453	-2.13188007	-0.13851105
C	1.18134517	-1.01364345	0.00585268
C	2.59070618	-1.20426931	0.04591450
C	3.13751559	-2.44911116	-0.44676130
C	-0.00308487	-4.56083360	-0.31008461
C	-1.35541750	-4.40918236	-0.14616677
C	-1.93847073	-3.10844525	-0.01326570
C	-1.09925357	-1.96742420	-0.06705805
C	-3.30922954	-2.90776789	0.27464668
C	-3.90105858	-1.65546423	0.38037700
C	-3.09694148	-0.52208289	-0.02784588
C	-1.68269140	-0.66996752	-0.07184679
C	-0.81532084	0.49039267	-0.08501587
C	0.60812514	0.31415419	0.08296585
C	-1.36533286	1.78388788	-0.16382411
C	-0.50967669	2.93158235	0.05347923
C	0.91727821	2.75492431	0.06929376
C	1.45208413	1.42734160	0.26587335
C	-1.03044928	4.23785642	0.27833869
C	-0.21242121	5.34882685	0.29156150
C	1.15470878	5.20762080	-0.01500663

C	1.69821265	3.94200343	-0.13597375
C	2.75611238	1.15105769	0.86394402
C	-2.74673742	1.88568944	-0.63406550
Cl	3.35126921	3.85984479	-0.72634008
C	3.37800266	2.07940575	1.73692501
C	4.51959122	1.76056848	2.45132957
C	5.07523901	0.47488361	2.33943012
C	4.47326013	-0.46009334	1.51460548
C	3.32260358	-0.15415200	0.74741205
C	-3.60165563	0.74005161	-0.55926955
C	-4.85785127	0.79254962	-1.21110508
C	-5.30465362	1.92617454	-1.86782959
C	-4.47408286	3.05587952	-1.93248853
C	-3.22029038	3.02112197	-1.34505471
C	-5.29463767	-1.60210062	1.09245329
C	-5.20806772	-2.49352362	2.36497380
C	-6.42077030	-2.17366603	0.20091760
C	-5.68675462	-0.20313933	1.61802465
C	4.56253220	-2.65471053	-1.05797081
C	4.38921621	-3.35572673	-2.43522354
C	5.43945565	-3.56956817	-0.17289814
C	5.31263678	-1.34077074	-1.36966023
H	2.64497306	-4.50818907	-0.81423772
H	0.43071071	-5.55670619	-0.43533103
H	-2.01078826	-5.28392499	-0.11726579
H	-3.90058375	-3.80037536	0.48722410
H	-2.09624270	4.35676460	0.46435850
H	-0.62873860	6.34062894	0.48235366
H	1.79100484	6.07998687	-0.17112650
H	2.91519800	3.05241293	1.89575758
H	4.95821485	2.49485059	3.13146947
H	5.94959924	0.19651920	2.93308838
H	4.85389391	-1.47924231	1.50358073
H	-5.46229742	-0.11040389	-1.24754488
H	-6.27402992	1.91991208	-2.37230345
H	-4.78745333	3.94210447	-2.48990698
H	-2.55659989	3.86872126	-1.49962332
H	-4.39253059	-2.16164436	3.02609562
H	-6.15231381	-2.42388375	2.92795233
H	-5.04493780	-3.55548795	2.13595124
H	-6.59191601	-1.57276204	-0.70435997
H	-7.37049407	-2.20500152	0.75988063
H	-6.19044601	-3.20021353	-0.12464963
H	-4.86701523	0.24812898	2.19844654

H	-5.96876233	0.50591984	0.83321457
H	-6.55324210	-0.30509847	2.29030816
H	3.73807249	-2.76657541	-3.09953454
H	5.37130614	-3.45674589	-2.92379487
H	3.96263167	-4.36514159	-2.35399595
H	5.65435019	-3.12516177	0.81009741
H	6.40653612	-3.76330330	-0.66551222
H	4.95346135	-4.54186420	0.00371511
H	4.67681481	-0.64175766	-1.93485060
H	5.67392678	-0.81560529	-0.47991323
H	6.19184298	-1.56935451	-1.99262258
**3c**			
C	-1.50893708	3.99192094	-0.53195005
C	-0.14150390	3.69302876	-0.32616853
C	0.22053085	2.33490836	-0.13950229
C	-0.79690432	1.35911115	0.04397640
C	-2.16321316	1.75455823	0.09601647
C	-2.52964827	3.05568436	-0.41817428
C	0.88788816	4.68637349	-0.37952282
C	2.20659394	4.34187696	-0.23539972
C	2.59592197	2.97316613	-0.07850416
C	1.59778797	1.96675012	-0.08696155
C	3.92870311	2.58080240	0.18968935
C	4.33360170	1.25821211	0.31821295
C	3.36325651	0.24582917	-0.04345043
C	1.98509588	0.59823985	-0.06635465
C	0.95755149	-0.42223980	-0.03283719
C	-0.42212086	-0.03611038	0.14668118
C	1.31176522	-1.78386716	-0.07734084
C	0.30151711	-2.78699407	0.18562425
C	-1.08547974	-2.40654950	0.20027945
C	-1.41644946	-1.01003278	0.36577798
C	0.63157253	-4.14496533	0.45896194
C	-0.33744709	-5.12481073	0.51854544
C	-1.67158545	-4.80068699	0.20070423
C	-2.02897137	-3.47589782	0.03169578
C	-2.65679383	-0.53318246	0.97151704
C	2.65373266	-2.10165899	-0.56469176
Br	-3.78763267	-3.17572417	-0.67621128
C	-3.39376317	-1.34270441	1.87254396
C	-4.46879661	-0.84741710	2.58982787
C	-4.83662479	0.50129510	2.45028992
C	-4.11731544	1.32123380	1.59728159
C	-3.03080176	0.83716421	0.82852117

C	3.66630769	-1.09030853	-0.54557804
C	4.88457123	-1.34253114	-1.22215082
C	5.14713141	-2.54860944	-1.84873219
C	4.16295698	-3.54951651	-1.85440020
C	2.94231231	-3.31651605	-1.24281281
C	5.71921996	1.01731407	1.00663375
C	5.78911475	1.94044128	2.25699267
C	6.89832647	1.39701205	0.08148258
C	5.91577761	-0.41185786	1.55999287
C	-3.91966250	3.45825122	-1.01209048
C	-3.66988639	4.11517765	-2.39968247
C	-4.64282891	4.49952464	-0.12700763
C	-4.85548444	2.26326949	-1.30061233
H	-1.74717646	5.01102155	-0.84207400
H	0.60189023	5.73186693	-0.52345559
H	2.98336529	5.11136822	-0.24127022
H	4.64776904	3.38256885	0.36785188
H	1.67128724	-4.40795987	0.64580972
H	-0.06780201	-6.15813624	0.74884827
H	-2.41870070	-5.58493568	0.07008960
H	-3.07245056	-2.36844692	2.04865995
H	-4.99948675	-1.49570274	3.29133507
H	-5.65521370	0.91586292	3.04393892
H	-4.34908221	2.38335544	1.56335885
H	5.61012050	-0.53677264	-1.30278356
H	6.09338717	-2.69755225	-2.37470638
H	4.33248038	-4.48923020	-2.38590720
H	2.16062183	-4.06463702	-1.35305011
H	4.94776701	1.74499932	2.93979076
H	6.72449640	1.74789612	2.80611972
H	5.77653388	3.00938973	2.00405923
H	6.96523881	0.75284339	-0.80766930
H	7.85318644	1.30662209	0.62509740
H	6.81168084	2.43669180	-0.27091539
H	5.04874655	-0.73006944	2.15951302
H	6.08280432	-1.16932360	0.78760546
H	6.79763266	-0.42026654	2.21990947
H	-3.11823682	3.43451487	-3.06662311
H	-4.63513113	4.34967094	-2.87581814
H	-3.10532247	5.05531639	-2.33520446
H	-4.91318291	4.09987507	0.86149152
H	-5.57463265	4.83142625	-0.61383394
H	-4.01550525	5.38969579	0.03728068
H	-4.33538001	1.47725739	-1.86988711

H	-5.27468686	1.79954064	-0.40196943
H	-5.70198842	2.61346459	-1.91213817

3d

C	-0.75912913	4.27333114	-0.56197357
C	0.56426649	3.80544259	-0.38547586
C	0.75458792	2.41734743	-0.16844625
C	-0.37377679	1.58561102	0.06739608
C	-1.67600461	2.15475388	0.14483885
C	-1.88806377	3.48025076	-0.39292275
C	2.07447399	1.87690151	-0.13325652
C	2.28500718	0.47113779	-0.07865758
C	1.13647274	-0.40780080	0.00156280
C	-0.17727128	0.15684270	0.19848168
C	1.71051205	4.65640856	-0.49206216
C	2.97755170	4.14963778	-0.36465391
C	3.19258597	2.74725707	-0.17282050
C	4.46956240	2.19545006	0.08572882
C	4.70466684	0.83630981	0.25052737
C	3.60701674	-0.05422180	-0.06516571
C	-3.23343111	4.04746477	-0.95449819
C	-2.94980484	4.62459376	-2.37060141
C	-4.33066133	2.98197052	-1.17215106
C	-3.77679610	5.19978105	-0.07912760
C	6.06135465	0.44172030	0.92535115
C	6.27355800	1.38627644	2.14368301
C	6.08315971	-0.98239418	1.52426879
C	7.26075176	0.63855646	-0.02967880
C	1.31179348	-1.80439116	-0.01004241
C	0.18644115	-2.66181578	0.29740628
C	-1.14072383	-2.10660401	0.32311697
C	-1.28254090	-0.67612162	0.46232307
C	0.34430619	-4.04153765	0.61302752
C	-0.74125224	-4.88435169	0.72406853
C	-2.02893554	-4.40196700	0.40886359
C	-2.21842207	-3.04827553	0.19968998
C	-2.43410426	-0.03177218	1.08749046
C	2.59373560	-2.30552147	-0.50420221
C	-3.24174262	-0.72249082	2.02639055
C	-4.21660359	-0.07658745	2.76711928
C	-4.40731912	1.30631941	2.61106346
C	-3.61694922	2.00956587	1.71722320
C	-2.63083613	1.37306224	0.92497977
C	3.72827867	-1.43306264	-0.52780105
C	4.89406968	-1.86175924	-1.20788604

C	4.99036728	-3.11041612	-1.79755876
C	3.88533873	-3.97525344	-1.76169208
C	2.71346318	-3.56736893	-1.14634404
I	-4.11673876	-2.52689906	-0.65515085
H	-0.87376458	5.30724278	-0.89301683
H	1.55683154	5.72567508	-0.66125852
H	3.84574763	4.81281473	-0.40945311
H	5.28918520	2.90243835	0.22770333
H	-3.89142990	4.97819657	-2.81976546
H	-2.25711392	5.47718995	-2.35789317
H	-2.52539230	3.85455153	-3.03342983
H	-5.14216341	3.42307519	-1.77249866
H	-4.77911668	2.61009221	-0.24537263
H	-3.94068612	2.11431347	-1.72651547
H	-4.05295593	4.86690668	0.93215953
H	-3.03316829	6.00462309	0.03085748
H	-4.67788188	5.63726390	-0.53982972
H	7.18753427	1.09216321	2.68380879
H	6.39285477	2.43994097	1.85645624
H	5.42825910	1.32227802	2.84643867
H	6.96912943	-1.08411571	2.17075887
H	6.13602976	-1.77985486	0.77642134
H	5.19402326	-1.16553554	2.14741991
H	7.22519571	-0.03340728	-0.89981040
H	7.30218359	1.67036717	-0.41238303
H	8.20665017	0.44044904	0.50077397
H	1.34516087	-4.43085358	0.79239472
H	-0.60337938	-5.93528568	0.98913896
H	-2.86429938	-5.09707174	0.31125459
H	-3.05328108	-1.77930531	2.21166923
H	-4.80463872	-0.63684105	3.49808719
H	-5.14270098	1.83548851	3.22229893
H	-3.70806206	3.09243980	1.66884097
H	5.71700689	-1.16043305	-1.32142986
H	5.90247246	-3.39664026	-2.32705547
H	3.92489722	-4.94497463	-2.26405698
H	1.84030469	-4.21121784	-1.22521518
**3e**			
C	2.34480625	-3.43947484	-0.51172373
C	0.95156144	-3.36322521	-0.27527262
C	0.37589137	-2.07746993	-0.11777336
C	1.22295943	-0.94406582	0.01630909
C	2.63613178	-1.10823227	0.04135046
C	3.19917851	-2.34451105	-0.45481515

C	0.09766876	-4.51214650	-0.27065653
C	-1.25644903	-4.38234015	-0.10109785
C	-1.86122908	-3.09062312	0.02213438
C	-1.04109526	-1.93653636	-0.04258425
C	-3.23514263	-2.90863377	0.30777352
C	-3.84813035	-1.66507387	0.39746821
C	-3.06224084	-0.52338797	-0.02324191
C	-1.64577001	-0.64928592	-0.06097383
C	-0.79728430	0.52447055	-0.08397828
C	0.62733475	0.37271656	0.09548782
C	-1.36769695	1.80726507	-0.19063274
C	-0.52974974	2.96809848	0.01769630
C	0.89572215	2.81456372	0.07901080
C	1.45243574	1.50060613	0.28245267
C	-1.06813154	4.27244597	0.20982614
C	-0.27017366	5.39980969	0.22544426
C	1.10475272	5.27255538	-0.03168589
C	1.68560719	4.00809782	-0.10653611
C	2.77034682	1.24970632	0.86281216
C	-2.74682876	1.87896608	-0.67095762
C	3.39060781	2.18540071	1.72858335
C	4.55352309	1.88751779	2.41784236
C	5.13033986	0.61386669	2.29120108
C	4.52902842	-0.33101181	1.47678549
C	3.35978091	-0.04512707	0.73179212
C	-3.58531401	0.72260124	-0.57583489
C	-4.84136710	0.74636463	-1.22950443
C	-5.30194015	1.86123614	-1.90861906
C	-4.48589381	2.99985441	-1.99635705
C	-3.23263062	2.99306764	-1.40665830
C	3.06242489	3.96492194	-0.51758949
N	4.15337230	4.01226037	-0.91405657
C	4.61744066	-2.52585945	-1.08880069
C	4.43079166	-3.22833914	-2.46379286
C	5.52221590	-3.42830451	-0.21919187
C	5.34242317	-1.20062209	-1.41206454
C	-5.24421010	-1.62619965	1.10525174
C	-5.14697306	-2.50189872	2.38785344
C	-6.35875850	-2.22569079	0.21746967
C	-5.65919158	-0.22753817	1.61384631
H	2.74088263	-4.41472760	-0.80128723
H	0.54758783	-5.50143458	-0.39074260
H	-1.89642684	-5.26797119	-0.06238376
H	-3.81233323	-3.80812285	0.52976659

H	-2.13994532	4.37963527	0.36834129
H	-0.71057876	6.38616486	0.38607062
H	1.72952743	6.15550845	-0.17839649
H	2.91203300	3.14680959	1.90897573
H	4.99221211	2.62946131	3.08911018
H	6.02245530	0.35124211	2.86506226
H	4.92775228	-1.34293812	1.45700573
H	-5.43412726	-0.16465367	-1.24921175
H	-6.27059937	1.83248658	-2.41358349
H	-4.80951609	3.86972017	-2.57322628
H	-2.57880630	3.84475689	-1.58092944
H	3.76106988	-2.64733505	-3.11668456
H	5.40596614	-3.31499060	-2.96847172
H	4.01954624	-4.24367102	-2.37683813
H	5.74564999	-2.98213686	0.76117179
H	6.48428628	-3.60502319	-0.72747634
H	5.05531466	-4.40894273	-0.03680084
H	4.68779182	-0.50864942	-1.96399329
H	5.71505163	-0.67096174	-0.52973848
H	6.21235262	-1.41610653	-2.05222119
H	-4.33742918	-2.15121133	3.04660730
H	-6.09305898	-2.43937006	2.94847305
H	-4.96875886	-3.56396950	2.17090359
H	-6.53739377	-1.63863156	-0.69546766
H	-7.30921662	-2.26545313	0.77453934
H	-6.11151964	-3.25227229	-0.09527795
H	-4.84876246	0.24182899	2.19303690
H	-5.94824338	0.46884105	0.82028663
H	-6.52700284	-0.33491349	2.28348614

Table S3 β_{eff} fitting results of (a) **3b** and (b) **3e**

β_{eff}	20.7uj	40.6uj	60.5uj
**3b**	2.27 cm/GW	1.5 cm/GW	1.15 cm/GW
**3e**	2.53 cm/GW	1.28 cm/GW	1.11 cm/GW

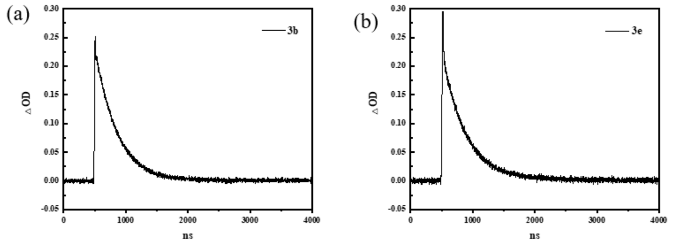

Figure S20 Decay trace of **3b** (a) and **3e** (b) at 480 nm.

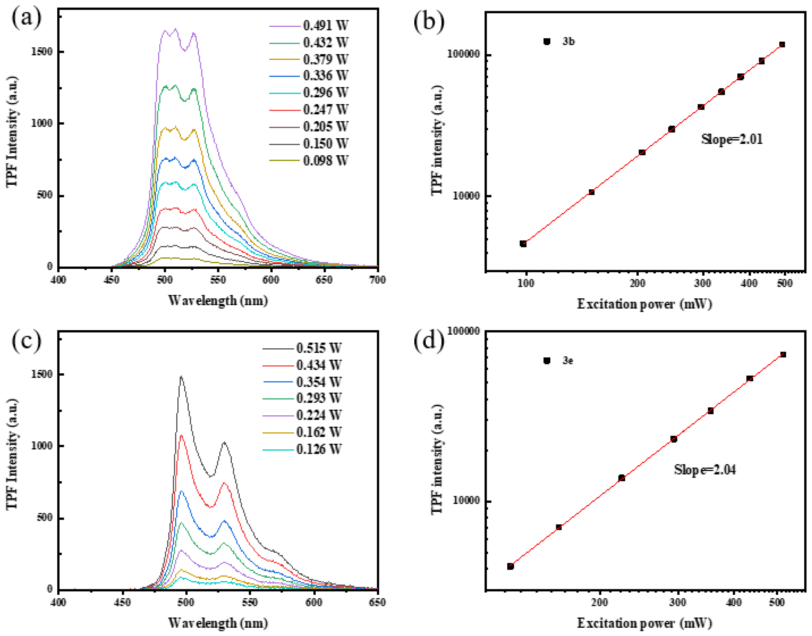

Figure S21 TPF intensity of **3b** (a) and **3e** (c). The linear fitting of power-dependent TPF intensity in logarithmic coordinates (b) and (d). The excitation wavelength is 800 nm.
